# University Students Adjusting to COVID-19 Stressors: Exploratory and Confirmatory Factor Analyses of the COVID-19 Stressors Questionnaire

**DOI:** 10.3389/fpsyg.2022.816961

**Published:** 2022-02-07

**Authors:** Minglee Yong, Hanna Suh

**Affiliations:** National Institute of Education, Nanyang Technological University, Singapore, Singapore

**Keywords:** COVID-19, factor analyses, depression, anxiety, stressor, adjustment, young adulthood

## Abstract

The psychological effects of COVID-19 have been documented in the past year, but scarce literature exists on the nature of COVID-19 stressors. Using a random split sample of 1199 young adult university students, results of exploratory factor analyses (EFA) identified a four-factor structure in the COVID-19 Stressors Questionnaire (C19SQ), which were labeled Resource Constraints, Social Restrictions, Future Uncertainty, and Health Concerns. This model was supported by a confirmatory factor analysis (CFA) when run on the other split sample of 1139 university students. Higher levels of COVID-19 stress were positively associated with anxiety and depressive symptoms and negatively associated with sleep duration, sleep quality, and the number of exercise days. COVID-19 stress also uniquely predicted poor university adjustment. This study demonstrated the link between COVID-19 stressors and mental and physical health symptoms, thus providing support for conceptualizing the psychological impact of the pandemic as adjustment problems for some individuals.

## Introduction

The COVID-19 pandemic is having devastating health, economic, political, social, and psychological impact on individuals and the society. These adverse outcomes are widespread and becoming increasingly pervasive, with its long-term effects still largely unknown. In terms of psychological impact, evidence is accumulating to show worsening mental health status in the different populations (Ettman et al., 2020; McCracken et al., 2020; Verma and Mishra, 2020). The adverse effects can be especially tumultuous for young adults since they are at a developmental stage that is associated with the most intense exploration of life’s possibilities including personal lives, relationships, and work (Arnett, 2000; Holmes et al., 2020). When the pandemic struck, many facets of human activities were curtailed, and social distancing measures have limited young people’s opportunities for life exploration and experiences, thus adding an additional layer of instability and uncertainty. It is thus important to explore how the pandemic is experienced by young adults.

Compared to rates of psychological distress before the pandemic, Essadek and Rabeyron (2020) found that prevalence of depression, anxiety, and distress were much higher than those normally observed in the student population during the pandemic. In a longitudinal study, Huckins et al. (2020) compared the mental health status of college students before and during the pandemic. Relative to previous academic terms, an increase in anxiety and depressive symptoms were reported in the first academic term impacted by COVID-19. In another longitudinal study surveying loneliness among United Kingdom adults, Bu et al. (2020) identified young adults (18–30 years) and being a student, among different risk factors, that heightened the risk of loneliness during the pandemic. Although informative, these studies primarily focused on the psychological effects of the pandemic, rather than the specific stressors associated with the effects. Hence, this study attempts to fill this gap in the literature, by identifying pandemic-related stressors that are salient for young adult population.

COVID-19 stressors are conceptualized as potential sources of stress. Consistent with the definition of stressors in the diagnostic criteria for Adjustment Disorder in the Diagnostic and Statistical Manual of Mental Disorders (DSM-5, American Psychiatric Association, 2013), stressors may be “a single event or multiple stressors,” and they may be “recurrent or continuous.” Applying this definition, the COVID-19 pandemic is associated with multiple continuous and pervasive stressors in the domains of health, family, school, and social life. With no clear endpoint of the pandemic in sight, the multiple COVID-19 stressors may become continuous. Consequently, these stressors are expected to impact student functioning until the stressors and their consequences are terminated or up until six months beyond that. Additionally, the “stressors may affect a single individual, an entire family, or a larger group or community.” In fact, COVID-19 stressors are affecting almost everyone in the world to different extent, some worse than others. Lastly, these COVID-19 stressors are also beyond one’s individual control given many necessary top-down changes imposed by governments and institutional entities. For university students COVID-19 stressors are likely to be additive to the stress inherently associated with the developmental task of adjusting to university life, such as being away from parents’ home, living at a new place, learning new things, completing internship, and graduating and transitioning to full-time employment. Taken together, COVID-19 stressors are considered to be conceptually similar to the stressors as defined in the criteria for Adjustment Disorder (American Psychiatric Association, 2013). Failure to adapt to these stressors can result in significant stress response that are associated with increased distress and significant impairment in daily functioning.

To understand the psychological impact of COVID-19, many studies developed instruments to measure symptoms of phobia, posttraumatic stress, fear, and anxiety (Ahorsu et al., 2020; Arpaci et al., 2020; Forte et al., 2020; Mertens et al., 2020; Petzold et al., 2020; Pérez-Fuentes et al., 2020; Varshney et al., 2020). Due to their focus on specific sets of symptomatology, many scales have a unidimensional structure (Ahorsu et al., 2020; Petzold et al., 2020; Pérez-Fuentes et al., 2020), which can be insufficient for understanding individuals’ experience during the pandemic. Furthermore, some researchers have argued that concepts of phobia, trauma, and posttraumatic stress cannot be applied to most people since the majority do not encounter life-threatening personal or health situations (Kazlauskas and Quero, 2020). On the other hand, many are affected for social, economic, and political changes that can become significant sources of stress. Furthermore, several studies did not evaluate the construct validity of their measures (Geldsetzer, 2020; Qiu et al., 2020; Zhong et al., 2020), thus limiting the utility of these instruments. Even fewer studies developed instruments to measure specific COVID-19 stressors and evaluated their impact on functioning (Kira et al., 2020; Zurlo et al., 2020; Ahuja, 2021). One study that did, investigated a sample of university students and identified three factors from their seven-item COVID-19 Student Stress Questionnaire (C19SSQ) (Zurlo et al., 2020). These were Relationships and Academic Life, Isolation, and Fear of Contagion. Although informative, this measure has limited content validity and unstable factor structure due to the small number of items for three factors. Other studies based on the general adult population showed more versatility in identifying COVID-19 stressors. They found factors related to routine disruption, future uncertainty, economic hardships, risk of infections, social problems, and systemic limitations (Kira et al., 2020; Ahuja, 2021). To address the limitations of current measures of COVID-19 stressors, this study attempted to develop more items to be representative of the COVID-19 stressors experienced by university students.

### Goals of Present Study

The goals of the study were to develop a measure of COVID-19 stressors relevant for university students, called the COVID-19 Stressors Questionnaire (C19SQ), and to analyze its factor structure. Another goal was to empirically demonstrate the relationship between the COVID-19 pandemic and the increased in psychological distress symptoms. Extending Zurlo et al. (2020)’s study, we considered a more comprehensive list of stressors, increased the number of items in C19SQ, and tested it with a larger sample. As predictive validity evidence was lacking in many COVID-19 measures reviewed here, we also considered the extent to which the C19SQ predicted university adjustment in students.

As part of the evaluation of the convergent validity of C19SQ, we hypothesized that COVID-19 stressors would be positively associated with anxiety and depressive symptoms. On the other hand, COVID-19 stressors would be negatively associated with a sense of belonging to the university, the overall adjustment to the university environment, and academic performance. Since stress has been consistently associated with a reduction of physical activity and sleep (Baglioni et al., 2010; Alvaro et al., 2013; Chekroud et al., 2018), we also hypothesized that COVID-19 stressors would be negatively associated with exercise and sleep. Group differences in terms of gender, race, and year of study were also investigated. Females and racial/ethnic minority groups were hypothesized to experience higher levels of COVID-19 stress (Horesh et al., 2020; Ruprecht et al., 2020).

## Materials and Methods

### Participants

A total of 2,345 undergraduate students from a university consented to participate in the online survey. This sample size was based on approximately 10% of the university population to ensure representativeness. A total of 92.3% of the participants completed the entire survey. The rest completed 4% to 92% of the survey. Data was collected between September and November 2020, during which a mask mandate was in place. After excluding seven students who were over 30 years or/and in their fifth year of study, the final sample comprised 1,309 females, 861 males, and 168 students who did not complete the section with the gender question. The mean age was 21.59 years (*SD* = 1.92 years, Range: 17 to 29 year). The racial composition based on those who reported the information was 86.8% Chinese, 3.9% Malay, 5.5% Indian, and 3.8% Others. In terms of the year of study, 562 students were in the first year; 627, in the second year; 596, in the third year; and 385, in the fourth year.

### Procedure

**FIGURE 1:**
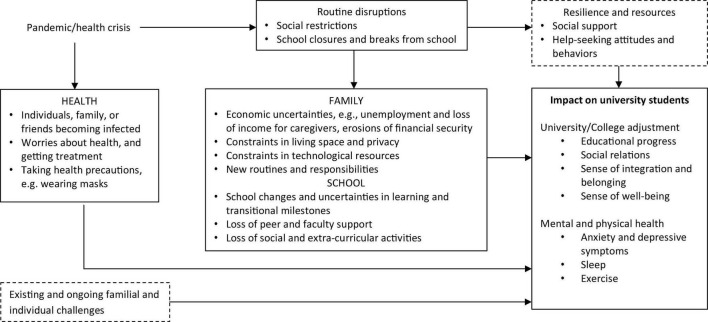
Direct and indirect effects of COVID-19 on university students. Factors enclosed in dotted boxes were not investigated in this study.

The framework in Figure 1 was developed to identify the direct and indirect effects of COVID-19 on young adults enrolled in universities across different domains of functioning, including health, family, school, and social life. Based on this framework, items were constructed to ensure adequate coverage across these domains. A literature search in the Psycinfo database and Google was also conducted in May 2020 to identify comparable measures of COVID-19 stressors. Relevant items not already in the scale were adapted for inclusion. In the next stage, the first author conducted a focused group discussion with a research staff and three psychology undergraduate students to brainstorm for more items, verify the face validity of the items, as well as refine and adapt them for cultural and language appropriateness. These procedures resulted in a 27-item C19SQ (Table 1). Even though the number of items in the initial pool was low, the use of a theoretical framework ensured good content validity.

**TABLE 1:** Standardized pattern coefficients for the two-factor, four-factor, and five-factor solutions in the first round of EFAs.

		Two-factor		Four-factor				Five-factor				
		1	2	1	2	3	4	1	2	3	4	5
	Abbreviated items (During the past few weeks, I worry about…)											
1	Myself getting sick from COVID-19	0.22	0.23		0.67				0.68			
2	Attending online lessons	0.33	0.23	0.23		0.31		0.51				0.31
3	Not being able to socialize with friends and relatives	0.71		0.68				0.39		0.52		
4	Changes in routines and schedules	0.55		0.48				0.55		0.27		
5	What is going to happen in the future	0.26	0.46	0.22	0.22		0.43		0.21		0.41	
6	Someone close to me becoming sick	0.25	0.35		0.61				0.62			
7	Having to take health precautions	0.48		0.29	0.40				0.39			
8	Not doing well in tests and exams		0.52				0.44				0.43	
9	Not being able to engage in recreational activities	0.71		0.73						0.66		
10	Going/commuting to school	0.24	0.36		0.32	0.41			0.31			0.39
11	Losing my freedom to travel to different places	0.59		0.55						0.49		
12	Arguments and conflicts at home		0.45			0.55					0.22	0.50
13	Completing my internship or degree		0.69			0.24	0.54				0.56	0.23
14	Whether school is a safe place because of COVID-19	0.24	0.42		0.57	0.21			0.56			
15	Space and privacy constraints at home		0.44			0.57					0.20	0.53
16	My future (e.g., education, career, and relationships)		0.70				0.80				0.83	
17	Not being able to see a doctor, counselor, or dentist	0.38	0.24		0.27	0.43			0.29			0.43
18	Not being able to attend outside school activities	0.81		0.80						0.83		
19	Not being able to participate in school activities and events	0.73		0.74						0.74		
20	Drifting away from friends socially	0.49		0.47						0.40	0.20	
21	Availability of food and supplies	0.34	0.26		0.32	0.39			0.34			0.40
22	Not being able to seek help from teachers and professors	0.30	0.33			0.45						0.44
23	More family responsibilities		0.47			0.59					0.20	0.55
24	Whether I have the skills and ability to cope with the future		0.72				0.78				0.79	
25	Money problems		0.57			0.25	0.42				0.45	0.23
26	Large number of COVID-19 cases	0.38	0.27		0.61				0.61			
27	Not having the technological resources to learn from home	0.29	0.24			0.48						0.47

*Coefficients < 0.2 are not reported. N = 1180. Two-factor solution: χ^2^(298) = 2354.79, p < 0.01; RMSEA = 0.08; CFI = 0.79; SRMR = 0.06. Four-factor solution: χ^2^(249) = 1056.70, p < 0.01; RMSEA = 0.05; CFI = 0.92; SRMR = 0.03. Five-factor solution: χ^2^(226) = 782.82, p < 0.01; RMSEA = 0.05; CFI = 0.94; SRMR = 0.03.*

An advertisement about the survey was sent to the emails of university students via their respective schools/colleges. Interested participants provided their consent and completed the survey online at their convenience. Data collection took place between October and December 2020. 75% of randomly selected participants received a $10 e-voucher at the end of the study. This study has received ethical approval from the Institutional Review Board at Nanyang Technological University.

### Measures

#### COVID-19 Stressors Questionnaire

The C19SQ initially comprised 27 items assessing the extent to which participants worry about health issues (6 items), family and home problems (6 items), school changes (7 items), social lives (5 items) and the future (3 items). Participants rated on a 4-point scale (1 = Not at all, 2 = A little, 3 = Sometimes, and 4 = A lot), the extent to which they were concerned about various aspects of life affected by the COVID-19 pandemic. High mean scores on this questionnaire indicated greater endorsement of COVID-19-related stress.

#### Patient Health Questionnaire-8

The Patient Health Questionnaire-8 (PHQ-8) which excluded the suicidal ideation item, was used in the present study to measure depressive symptoms. Participants self-reported about their experience of eight symptoms on a 4-point scale (1 = Not at all, 2 = A little, 3 = Sometimes, and 4 = A lot). The scale anchors were labeled differently as the original scale to facilitate consistency across various questionnaires in the survey. Higher mean scores on this scale indicated higher levels of depressive symptoms. The internal consistency estimates for this scale in the present study were 0.85 and 0.84 for males and females, respectively.

#### Generalized Anxiety Disorder-7

The Generalized Anxiety Disorder-7 (GAD-7) assesses seven symptoms of anxiety and their severity. Participants rated themselves on a 4-point scale (1 = Not at all, 2 = A little, 3 = Sometimes, and 4 = A lot), which was also labeled differently from the original scale. Higher mean scores indicated higher levels of anxiety symptoms. The internal consistency estimates were 0.90 and 0.89 for males and females, respectively.

#### Social Integration

The four-item Social Integration (SI) scale was used to measure students’ sense of belonging to the university. This scale was adapted from the National Survey of Student Engagement (Kuh, 2001). Examples of items were “I feel that I am part of [university name]” and “I feel comfortable being myself at [university name].” The internal consistency estimates were 0.87 for males and 0.88 for females.

#### College Adjustment Questionnaire

University adjustment was measured by the 14-item College Adjustment Questionnaire (CAQ) which includes three domains of college adjustment, namely, Educational, Relational, and Psychological. A 5-point scale was used by participants to indicate how true certain statements about college or university experiences apply to them at the time of the survey (1 = Very inaccurate, 2 = Moderately inaccurate, 3 = Neither inaccurate or accurate, 4 = Moderately accurate, and 5 = Very accurate). Higher mean scores indicate better college adjustment. The internal consistency estimates for the overall scale were 0.88 for both males and females.

#### Grade Point Average

Given the option of 10 Grade Point Average (GPA) bands (1 = 0 to 0.49, 2 = 0.5 to 0.99, 3 = 1.0 to 1.49, 4 = 1.5 to 1.99, 5 = 2.0 to 2.49, 6 = 2.5 to 2.99, 7 = 3.0 to 3.49, 8 = 3.5 to 3.99, 9 = 4.0 to 4.49, and 10 = 4.5 to 5.0), participants reported their latest GPA scores as a measure of their academic performance.

#### Sleep and Exercise

Participants reported on the number of hours of actual sleep per night, as well as the quality of their sleep. These items were adapted from the Pittsburg Sleep Quality Index (Buysse et al., 1989). Additionally, they also reported the number of days they exercise per week for more than 10 min each time.

### Analyses

Principal component and parallel analyses (O’Connor, 2000) were conducted in SPSS Version 25 on a randomly split half of the sample. These results were compared, and the scree plot was inspected to determine the number of factors to retain in the subsequent exploratory factor analyses (EFAs). Based on theoretical considerations, individual items were reviewed in terms of how they were related to other items and common COVID-19 stressors identified in the literature. EFAs based on different number of factors were tested and compared in Mplus Version 8.4 (Muthén and Muthén, 1998–2017), using the maximum likelihood estimator that generates standard errors robust to non-normality and non-independence of observations (MLR). The Geomin oblique rotation was used to allow for correlation among factors. In general, items with rotated factor loadings < 0.4 were excluded from further analyses while maintaining appropriate scale length. Models with fewer cross-loadings were preferred since they would exhibit lower factor intercorrelations and were more likely to approximate a simple structure. Model fit was evaluated based on RMSEA was ≤ 0.06, CFI ≥ 0.95, or SRMR ≤ 0.08 (Hu and Bentler, 1999). Items and factors were continuously reviewed for their theoretical relevance in multiple runs of EFAs.

After the final EFA model was determined, a confirmatory factor analysis (CFA) was run on the other half sample to confirm the factor structure. Preliminary analyses showed no significant differences between the two randomly split samples on all the dependent variables. Both modification indices and theoretical considerations were used to help with improvement of model fit in re-runs of the CFAs. Although reported, Chi-square was not used as an index of model fit since trivial differences between the sample and estimated population covariance matrices often led to significant Chi-square with large samples (Tabachnick and Fidell, 2007). Factor reliability was evaluated based on Cronbach’s alpha, with values > 0.70 being acceptable.

For convergent validity investigations, the associations between C19SQ and other relevant self-report variables were evaluated based on Pearson’s product-moment correlation coefficients. Scores for the factors were based on means of items that load on those factors confirmed by the CFA results. Hierarchical multiple regression analysis was then used to assess the incremental validity of C19SQ in predicting college adjustment of students during the current pandemic, beyond the measures of academic achievement, mental health symptoms, and social integration. These covariates were selected since they were expected to be significantly correlated with the outcome measure. Hence, CAQ was regressed on GPA, SI, PHQ, and GAD in the first block of the regression model; C19SQ was then entered in the second block. The change in variance (Δ*R*^2^) in the second block provided evidence about the incremental validity of the C19SQ. In the last series of analyses, groups of students based on gender, race, and year of study were compared in terms of their C19SQ scores using a *t*-test or a non-parametric test of difference.

Missing data in the dataset was minimal (3.46%) and was accommodated using full information maximum likelihood in Mplus for the EFA and CFA analyses. As 19 and 21 cases were missing on all C19SQ items, they were excluded from the EFAs and CFAs, respectively. In correlational analyses, all available data pertaining to the measures were used. Due to listwise deletion, 178 cases (7.61%) were excluded from the regression analyses. Overall, statistical power was reasonably assured given the big sample size.

## Results

A principal components analysis (PCA) conducted on about half the sample (*n* = 1199) indicated that five components had eigenvalues > 1.0. According to a parallel factor analysis which was compared with the PCA results, four factors should be retained. Examining the scree plot, only two factors were at and above the “elbow”. Given these results, EFAs were conducted for two-factor, four-factor, and five-factor solutions (Table 1). Comparing the pattern matrices for the different solutions, the two-factor solution had nine items with low pattern coefficients of < 0.4, which would entail removing too many items in the next step. On the other hand, the four- (Item 2 and 21) and five-factor (Item 7 and 10) solutions had only two such items each. Additionally, based on maximum likelihood estimates with robust standard errors, the two-factor model did not fit the data well while the other models showed better fit. Hence, the two-factor solution was no longer considered in subsequent analyses.

In the four- and five-factor solutions, the patterns of loadings were largely similar. In both solutions, Item 1, 6, 7, 14, and 26 loaded on one factor; 3, 9, 11,18, 19, and 20 loaded on another factor; 5, 8, 13, 16, 24, and 25 loaded on another factor; and 10, 12, 15, 17, 22, 23, and 27 loaded on another factor. On the other hand, Item 2, 4, and 21 loaded differently in the two solutions. In the five-factor solution, Item 2 and 4 loaded on a fifth factor. Item 21 which was about the availability of food and supplies, appeared to load better in the five-factor solution, together with items about home and resources. On the other hand, it was together with other items about health in the four-factor solution, which was not as appropriate. Overall, the consistent pattern of loadings of all other items indicate stability in four of the factors in the solutions.

In the second round of EFAs, items with low pattern coefficients were removed and the four- and five-factor solutions were re-run. After dropping Item 2 and 21 for the four-factor model and Item 7 and 10 for the five-factor model, both the revised models showed improvements in fit. There were fewer cross-loadings in the four-factor model (6 items) than the five-factor model (9 items). 15 out of 25 items in the four-factor model had loadings > 0.5, compared to 14 out of 25 items in the five-factor solution.

In the third round of EFAs, additional items, 10 and 22 were dropped in the four-factor model, while item 20 was dropped from the five-factor model, due to the pattern coefficients being < 0.4. Comparing between the 23-item four-factor model and the 24-item five-factor model, both appeared to achieve adequate fit to the data. However, the five-factor model continued to have 9 items cross-loading on more than one factor while the four-factor model reduced to five items. Having only two indicators for the fifth factor might risk problems of underidentification and non-convergence. Further analyses also indicated poor reliability of the fifth factor (α = 0.60) and poor discrimination between this factor and another factor due to their high latent factor correlation (0.83). Hence, the five-factor model was rejected, and the four-factor model was retained (Table 2). No more items were excluded even though three pattern coefficients in the four-factor model were < 0.4, to avoid shortening the scale further. As shown in Table 2, The four factors were labeled Health Concerns (Item 1, 6, 7, 14, and 26), Social Restrictions (Item 3, 4, 9, 11, 18, 19, and 20), Future Uncertainty (Item 5, 8, 13, 16, 24, and 25), and Resource Constraints (Item 12, 15, 17, 23, and 27).

**TABLE 2:** Standardized pattern coefficients for the final four-factor solution after excluding Item 2, 10, 21, and 22.

	Abbreviated item (During the past few weeks, I worry about…)	Resource constraints	Social restrictions	Future uncertainty	Health concerns
1	Myself getting sick from COVID-19				0.69
3	Not being able to socialize with friends and relatives		0.65		
4	Changes in routines and schedules		0.50		
5	What is going to happen in the future			0.42	0.22
6	Someone close to me becoming sick				0.62
7	Having to take health precautions		0.30		0.41
8	Not doing well in tests and exams			0.44	
9	Not being able to engage in recreational activities		0.73		
11	Losing my freedom to travel to different places		0.54		
12	Arguments and conflicts at home	0.68			
13	Completing my internship or degree	0.21		0.53	
14	Whether school is a safe place because of COVID-19				0.57
15	Space and privacy constraints at home	0.68			
16	My future (e.g., education, career, and relationships)			0.82	
17	Not being able to see a doctor, counselor, or dentist	0.35			0.27
18	Not being able to attend outside school activities		0.82		
19	Not being able to participate in school activities and events		0.75		
20	Drifting away from friends socially		0.46		
23	More family responsibilities	0.60			
24	Whether I have the skills and ability to cope with the future			0.80	
25	Money problems	0.25		0.39	
26	Large number of COVID-19 cases				0.62
27	Not having the technological resources to learn from home	0.37			

*Coefficients < 0.2 are not reported. χ^2^(167, n = 1180) = 638.50, p < 0.01; RMSEA = 0.05; CFI = 0.94; SRMR = 0.03.*

**FIGURE 2:**
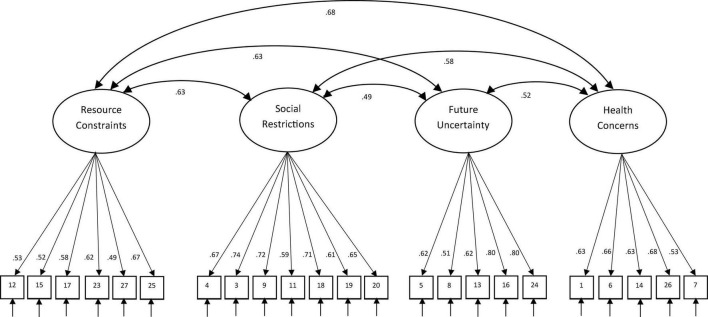
Standardized factor loadings and latent factor correlations for the final 23-item four-factor CFA model.

Using the second split sample (*n* = 1139), a confirmatory factor analysis on the 23 items showed that a four-factor solution did not attain an adequate fit, χ^2^(224, *n* = 1118) = 1292.40, *p* < 0.05, RMSEA = 0.06, CFI = 0.88, and SRMR = 0.06. Based on the modification indices, Item 18 and 19 were allowed to covary since both were related to school activities. problems experienced in the home setting. Next, considering that Item 25 was about money problems due to COVID-19 and could be consistent with the Resource Constraints factor, this item was re-specified to load on Resource Constraints. Last, Item 12 and 15 were also allowed to covary since both were related to home issues. With these stepwise re-specifications, the model as illustrated in Figure 2, attained a good fit based on RMSEA and SRMR criteria, χ^2^(222, *n* = 1118) = 983.17, *p* < 0.05, RMSEA = 0.055, CFI = 0.90, and SRMR = 0.05. According to the latent factor correlations, the significant correlations among the five factors were mostly in the medium-strong range (0.52–0.68). The reliability estimates of the factors based on the Cronbach alpha were 0.76 for Resource Constraints (6 items); 0.86 for Social Restrictions (7 items), 0.80 for Future Uncertainty (5 items), and 0.77 for Health Concerns (5 items). The estimate for the 23-item scale was 0.90.

To establish convergent validity with related variables, the C19SQ was correlated with PHQ, GAD, SI, CAQ, GPA, as well as sleep and exercise indicators. Table 3 shows the correlation matrix among these variables by gender. The C19SQ total score showed large and positive correlations with PHQ and GAD. The expected negative correlation between C19SQ and CAQ were also large and significant. With SI, it showed small to moderate negative correlation. C19SQ showed small to moderate negative correlations with sleep hours per night and sleep quality. Lastly, C19SQ was significantly correlated with the number of days of exercise per week for males but not for females. C19SQ and its factors were generally not significantly associated with GPA but GPA was positively associated with CAQ.

**TABLE 3:** Correlation matrix of measured variables.

	Females *n* = 1309 *M* (*SD*)	Males *n* = 861 *M* (*SD*)	1.	2.	3.	4.	5.	6.	7.	8.	9.	10.	11.	12.	13.
1.C19SQ	2.05 (0.52)	1.99 (0.54)		0.78**	0.82**	0.77**	0.74**	0.47**	0.51**	−0.43**	–0.002	−0.14**	−0.17**	−0.24**	–0.05
2.CRes	1.73 (0.60)	1.64 (0.57)	0.80**		0.48**	0.54**	0.46**	0.43**	0.44**	−0.34**	–0.01	−0.17**	−0.14**	−0.23**	–0.05
3.CSocial	1.95 (0.68)	1.99 (0.72)	0.84**	0.54**		0.45**	0.50**	0.32**	0.31**	−0.33**	–0.01	–0.04	−0.12**	−0.16**	0.02
4. CFuture	2.68 (0.73)	2.54 (0.76)	0.78**	0.57**	0.49**		0.44**	0.50**	0.56**	−0.50**	–0.001	−0.21**	−0.16**	−0.23**	−0.10**
5.CHealth	1.94 (0.65)	1.86 (0.66)	0.77**	0.53**	0.54**	0.48**		0.23**	0.28**	−0.16**	0.03	–0.03	−0.11**	−0.15**	–0.05
6.PHQ	2.20 (0.63)	2.03 (0.63)	0.49**	0.49**	0.34**	0.49**	0.26**		0.73**	−0.53**	–0.03	−0.26**	−0.22**	−0.38**	−0.12**
7.GAD	2.24 (0.74)	2.00 (0.73)	0.54**	0.52**	0.37**	0.55**	0.31**	0.73**		−0.49**	–0.03	−0.19**	−0.20**	−0.35**	−0.09**
8.CAQ	3.01 (0.68)	3.14 (0.68)	−0.43**	−0.38**	−0.31**	−0.49**	−0.19**	−0.55**	−0.49**		0.23**	0.40**	0.23**	0.32**	0.16**
9.GPA	7.80 (2.24)	8.08 (2.10)	–0.01	–0.07	0.03	–0.05	0.04	–0.05	–0.05	0.26**		0.08**	0.08**	0.06*	0.04
10.SI	2.79 (0.55)	2.74 (0.61)	−0.18**	−0.19**	–0.07	−0.26**	−0.07*	−0.31**	−0.26**	0.47**	0.11**		0.07*	0.21**	0.10**
11.SleepD	2.63 (0.93)	2.70 (0.92)	−0.18**	−0.21**	−0.10**	−0.18**	−0.12**	−0.25**	−0.21**	0.26**	0.12**	0.10**		0.45**	0.07*
12.SleepQ	2.67 (0.67)	2.68 (0.69)	−0.27**	−0.31**	−0.16**	−0.29**	−0.12**	−0.48**	−0.37**	0.39**	0.06	0.24**	0.43**		0.12**
13.Exer	1.71 (1.84)	2.30 (1.98)	−0.12**	−0.11**	–0.05	−0.14**	−0.12**	−0.21**	−0.17**	0.18**	0.02	0.10**	0.06	0.14**	

* p < 0.05, ** p < 0.01. C19SQ = 23-item COVID-19 Stressors Questionnaire, CRes = C19SQ Resource Constraints subscale, CSocial = C19SQ Social Restrictions subscale, CFuture = C19SQ Future Uncertainty subscale, CHealth = C19SQ Health Concerns subscale, PHQ = Patient Health Questionnaire-8, GAD = General Anxiety Disorder-7, SI = Social Integration, CAQ = College Adjustment Questionnaire, GPA = Grade Point Average, SleepD = Sleep Duration, SleepQ = Sleep Quality, Exer = Exercise. Results for females and males are above and below the diagonal, respectively.

To test the effects of C19SQ on college adjustment over and beyond the existing psychological distress that students may already experience, two multiple regression models were tested. In the first regression model, the predictors, PHQ, GAD, SI, and GPA, accounted for a significant amount of variance in CAQ, *R*^2^ = 0.43, *F*(4, 2155) = 412.74, *p* < 0.01, *R*^2^_adjusted_ = 0.43. All predictors remained uniquely predictive of CAQ, with standardized regression coefficients in the medium range (0.20 < effect sizes < 0.30). When C19SQ was entered into the second block [β = -0.19, *t*(2154) = 10.02, *p* < 0.01, *pr*^2^ = 0.04], it accounted for Δ*R*^2^ value of 0.02, Δ*F*(1, 2154) = 96.50, *p* < 01, which might be considered a significant portion of incremental variance beyond the effects of the first block measures in the stringent model. The assumptions for running these multiple regression models, including normality of the CAQ, homoscedasticity of residues, and independence of responses were evaluated to be tenable.

Tests of groups differences on C19SQ indicated that females scored higher on C19SQ than males [Mean difference = 0.05, *t* (2168) = 2.48, *p* < 0.05, *d* = 0.11], though this might be considered a small effect. Test of homogeneity of variances across racial/ethnic groups indicated a significant difference. Hence, a non-parametric test of difference was conducted. A Kruskal-Wallis test showed that there was a statistically significant difference in C19SQ among the different race/ethnic groups. Dunn’s paired comparisons with significance adjusted by Bonferroni correction for multiple tests indicated that that Malay students (Mean rank = 1,213, *p* < 0.05) and Indian students (Mean rank = 1,265, *p* < 0.01) scored significantly higher on C19SQ compared to their Chinese counterparts (Mean rank = 1,022). No other comparisons were significant. No significant differences were found among students from different years of study.

## Discussion

The present study developed a university student-specific COVID-19 stressor scale (C19SQ), identified its factor structure, and ensured convergent and predictive validity of this newly developed scale. Findings from this study allow for a better understanding about the salient COVID-19 stressors experienced by university students. Results of the EFAs and CFA identified four underlying factors, namely, Resource Constraints, Social Restrictions, Future Uncertainty, and Health Concerns.

The specific factor of Resource Constraints identifies a significant area of stress for university students that includes limitations in technological resource, financial difficulties, difficulties accessing medical services, space and privacy constraints at home, as well as increased family responsibilities. This is consistent with other studies which found that university students lost employment opportunities and had to cope with financial insecurity during this pandemic (Essadek and Rabeyron, 2020; Kecojevic et al., 2020). As a result of sheltering-in-place and quarantine measures, some students also experienced constraints in living space and privacy which can lead to increased interpersonal conflicts (Alzueta et al., 2021). When healthcare systems are strained by high number of infection or social distancing measures, students may also be affected by limited access to health services. Hence, as a result of resource constraints, students may struggle to find and harness the resources they need for their well-being and development during this pandemic.

The factor of Social Restrictions also represents a significant stressor for university students who are considered emerging adults (Arnett, 2000). At this developmental stage, students are engaged in extensive exploration of life possibilities, including social connectedness and relationships with others. With the on-going pandemic, the range of social experiences is suddenly limited and may not become available again, such as internship or fieldwork experience, university-level sports competitions, and convocations. In addition to the loss of social opportunities, diminished social support from peers, faculty, and staff may also affect university adjustment. In fact, when schools closed, students who did not move back to their parents’ homes, were found to be more vulnerable to stress, probably due to the loss of both formal and informal forms of social and academic support that are typically available in a university environment (Husky et al., 2020). Social restrictions can also increase feelings of loneliness, which in turn, are predictive of depressive and anxiety symptoms (Bu et al., 2020; Groarke et al., 2020). Hence, at a developmental stage when emerging adults typically form more extensive networks of social relations, social restrictions during the pandemic can be a significant source of stress for university students unable to develop or access their social network of support.

Future Uncertainty also emerged as another source of stress for university students who are at a developmental stage already characterized by change and uncertainty (Arnett, 2000). Not being able to tolerate uncertainty has been found to be associated with increased levels of generalized anxiety and other emotional distress (Boswell et al., 2013). Intolerance of uncertainty may be due to negative beliefs and appraisals about threat and coping, which results in maladaptive responses, in terms of emotions, cognitions, and behaviors (Freeston et al., 1994). Given so much uncertainty about the pandemic, questions abound concerning the effectiveness and side effects of vaccines, the recovery of the economy, and the safety of travel. Besieged by a continuous stream of misinformation on the internet, people are already coping with more uncertainty than usual (Rettie and Daniels, 2020). Worse for university students, uncertainty about their future is now further heightened by unknowns regarding how the pandemic will pan out and the impact on their future plans and aspirations.

During a pandemic, the factor of Health Concerns represents a ubiquitous source of stress for individuals and their families dealing with a heightened risk of infection, ill health, and even, death. Living in areas with high infectious spread of COVID-19 has been found to be a predictive factor of stress and depressive symptoms in a sample of university students (Tang et al., 2020). Many may be concerned about the highly contagious nature of the COVID-19 virus, the lack of good medical knowledge about the virus and its treatment, and the potential for health system to be quickly overwhelmed by high number of infections. This stress is further compounded when public health information is not consistent, or when there is poor adherence to public health advisories. Additionally, the prevalence of misinformation on social media is likely to increase worries about health risks. This may partly explain why increased phone use or media exposure by university students during the pandemic has been associated with higher levels of anxiety and depressive symptoms (Huckins et al., 2020; Kecojevic et al., 2020; Ma et al., 2020). Even though having health concerns may be typical during a pandemic, high and continuous levels of health concerns can become a source of stress and anxiety, resulting in indiscriminate avoidance of activities and difficulties coping with daily social and academic demands.

In terms of convergent validity, the C19SQ as an entire scale, exhibited large and significant positive associations with anxiety and depressive symptoms. Consistent with other studies, students who endorsed higher levels of COVID-19-related concerns, were more likely to experience anxiety or depressive symptoms. While other studies demonstrated higher rates of symptoms after the onset of the pandemic (Essadek and Rabeyron, 2020; Huckins et al., 2020), the results of this study more directly linked COVID-19 stressors, as measured by the C19SQ, with anxiety and depressive symptoms. At the same time, COVID-19 stress was negatively associated with social integration, university adjustment, and exercise and sleep patterns. Students experiencing more COVID-19 stress reported feeling lower sense of belonging to the university and adjusting more poorly to university life. Furthermore, they were also exercising and sleeping less, and having poorer sleep quality. Even after controlling for mental health symptoms, the sense of social integration, and academic results, COVID-19 stress remained a unique and significant predictor of university adjustment. Despite the small effect size, the findings suggest that COVID-19 stress contributed to poor university adjustment.

In terms of the physical health effects of COVID-19 stress, this study found that an increase in stress was associated with a reduction in the number of days students exercised and the number of sleep hours, as well as poorer quality sleep. The curtailment of social activities due to social distancing measures, including restrictions in the use of sporting and recreational facilities, may have expectedly led to a reduction of physical activity and a concomitant increase in sedentary behavior (Giustino et al., 2020; Hall et al., 2020; Huckins et al., 2020). Given the reciprocal association between physical activity and mental health (Chekroud et al., 2018), a reduction of physical activity and exercising during COVID-19 is likely to reciprocally contribute to poorer mental health (Tang et al., 2020), with the effect expected to be more pronounced for those who usually lead a sedentary lifestyle (Lesser and Nienhuis, 2020). In general, university students are more susceptible to stress, anxiety, and depressive symptoms, as well as poor sleep and sleep behaviors, thus explaining how these are problems are consistently comorbid with one another (Alvaro et al., 2013; Peach et al., 2016), including during this pandemic.

### Group Differences in the Effects of COVID-19 Stressors

In this study, female students reported experiencing more COVID-19-related stressors, as well as anxiety, and depressive symptoms. These results are consistent with other COVID-19 studies (Essadek and Rabeyron, 2020; Fitzpatrick et al., 2020; Rettie and Daniels, 2020; Tang et al., 2020), as well as epidemiological studies documenting higher rates of depression and anxiety among female adults. Minority groups, namely Malay and Indian students, were also found to experience higher levels of COVID-19 stress. Disparities in the experience of COVID-19 among groups from different racial/ethnic or socioeconomic backgrounds may operate at three levels, namely, exposure, susceptibility, and treatment access (Blumenshine et al., 2008). The relevance of these factors for understanding the increased in COVID-19 stressors among Malay and Indian university students in Singapore remain to be investigated. Lastly, no difference was found in the experience of COVID-19 stressors among university students from different years of study. This result contrasts with another study which found that senior year students were more likely to experience depressive and anxiety symptoms (Ma et al., 2020). In sum, findings of groups differences on the C19SQ are generally consistent with other COVID-19 studies on gender and minority group differences.

### Implications

The current evidence linking COVID-19 stressors to the higher risk of psychological symptoms and poorer adjustment and functioning in the university supports the understanding of the psychological impact of COVID-19 as stress responses or adjustment reactions. Current university students not only have to deal with typical stressors related to developmental events during the stage of emerging adulthood, they also have to cope with additional COVID-19 stressors during this pandemic. Despite the current increase in challenges, it is expected that symptoms and functioning will improve when COVID-19 stressors are “terminated” or when individuals show increased coping ability to deal with the stress. Given that the COVID-19 pandemic is still ongoing, some stressors may continue to be impactful. For some individuals, their stress reactions may become chronic, putting them at a higher risk of comorbid physical and mental health challenges.

The identification of the specific COVID-19 stressors in terms of resource constraints, social restriction, future uncertainty, and health concerns, helps us to understand the operating mechanisms that result in the psychological effects of COVID-19. This will allow service providers to target these specific factors to mitigate the effects of COVID-19. Hence, it is recommended that supporting the mental well-being of university students includes (1) identifying practical resources to help them cope with daily functioning, (2) encouraging them to stay connected with others in creative ways and develop new social routines, (3) increasing their tolerance for future uncertainty, and (4) discussing their health concerns and referring them to accurate and reliable sources of information. Additionally, it is important to help students understand the factors contributing to their distress and validate their experience of change and uncertainty during this unprecedented time.

### Limitations and Strengths

One of the limitations of the study may be that the initial item pool of 27 items was small, thus limiting content validity. However, this problem was mitigated with the use of a theoretical framework to guide item generation. To achieve a stable four-factor structure, only four items were excluded, reflecting the good quality of the items. Additionally, there are at least five items loading on each factor, which contributed to the reliability of each factor. Secondly, even though items were generated based on the broad domains of health, family, school, and social life, results from the factor analyses might not appear to support COVID-19 stress as “domain-specific” as first theorized. However, it should be noted that health and social life are related to Health Concerns and Social Restrictions, respectively. Furthermore, the interpretation and labeling of the factors were based on the psychological relevance and applicability of the constructs, such as Future Uncertainty and Resource Constraints. Thirdly, the current study used a cross-sectional dataset which would limit conclusions about the causal impact of COVID-19 stressors. Longitudinal monitoring of the effects of COVID-19 is thus expected to provide stronger evidence for understanding these effects. The fourth limitation may concern the generalizability of the results to university students in other countries since the experience of COVID-19 may differ based on different responses of countries and communities when faced with fluctuating infectious spread of COVID-19. Hence, the C19SQ should be evaluated for measurement invariance when used with other populations in future research.

This study has several strengths which include a large sample size of over 2,000 students, thus providing power for the analyses conducted, and allowing for more accurate estimates of effects. The study has also taken an additional step beyond the identification of symptomatology associated with COVID-19 by demonstrating the predictive validity of the C19SQ with respect to university adjustment, over and above other predictors of university adjustment in a stringent test of the effects of COVID-19.

The emergence of psychological symptoms as a result of the pandemic may not be unexpected given that most people are trying to adjust and adapt to multiple stressors. While our understanding about the psychological impact of the pandemic has increased rapidly, the long-term effects of COVID-19 are still unknown and continue to be investigated. Given that adjustment disorder is one possible way that individuals could be affected by COVID-19, it is hoped that the clarification of the nature of stressors in this study can guide further understanding, prediction, and mitigation of the psychological effects of COVID-19.

## Data Availability Statement

The raw data supporting the conclusions of this article will be made available by the authors, without undue reservation.

## Ethics Statement

The studies involving human participants were reviewed and approved by Nanyang Technological University IRB. Written informed consent for participation was not provided by the participants’ legal guardians/next of kin because: All participants were university students over 17 years old. NTU IRB waived the requirement for parental consent.

## Author Contributions

MY: methodology, formal analysis, investigation, writing – original draft preparation, and resources. MY and HS: conceptualization, writing – review and editing, and funding acquisition. Both authors contributed to the article and approved the submitted version.

## Conflict of Interest

The authors disclosed receipt of the following financial support for the research, authorship, and/or publication of this article: This work was supported by the NIE Planning Grant, Ministry of Education, Singapore [PG 08/20 YML].

## Publisher’s Note

All claims expressed in this article are solely those of the authors and do not necessarily represent those of their affiliated organizations, or those of the publisher, the editors and the reviewers. Any product that may be evaluated in this article, or claim that may be made by its manufacturer, is not guaranteed or endorsed by the publisher.
